# Poppy Seed Muffins Causing Unexpected Opioid Positives in Patients Receiving Opioid Substitution Therapy: A Case Series

**DOI:** 10.1192/bjo.2026.11760

**Published:** 2026-06-30

**Authors:** Mohammad Ali

**Affiliations:** Addictions Recovery Community (ARC-MK), Milton Keynes, United Kingdom

## Abstract

**Aims:**

Opioid substitution therapy (OST) with methadone or buprenorphine is a cornerstone of treatment for opioid use disorder, with urine toxicology screening routinely used to monitor adherence and detect illicit opioid use. Unexpected positive opioid results can lead to clinical confusion, unnecessary dose adjustments, and potential strain on the therapeutic alliance. Dietary sources, particularly poppy seed–containing foods such as muffins, bagels, or pastries, have been reported to cause transient, false-positive opioid urine screens due to the morphine and codeine content of the seeds.

Despite being well-documented in pharmacology literature, awareness of this phenomenon in clinical addiction psychiatry remains limited, and it may lead to unnecessary interventions or patient distress. This case series aims to describe instances of poppy seed–induced false-positive opioid screens in patients receiving OST, highlight the clinical impact, and provide practical guidance for recognizing and managing this rare but important interaction.

**Methods:**

Three adult OST patients (aged 28–45)–two on methadone, one on buprenorphine–were identified after routine urine toxicology unexpectedly tested positive for opiates (negative for 6-MAM). All had stable adherence, no illicit opioid use, and no recent dose changes.

History revealed recent consumption of poppy seed–containing baked goods: two muffins and one bagel. No other medications, supplements, or substances were reported. Patients were counseled, and repeat testing 48–72 hours later was negative for opioids, confirming dietary exposure. No withdrawal, overdose, or adverse events occurred, and OST adherence remained unchanged.

**Results:**

These cases illustrate a clinically important but under-recognized phenomenon: dietary intake of poppy seed–containing foods can produce transient false-positive opioid results in patients receiving OST. All three patients demonstrated positive urine screens despite stable adherence, no illicit opioid use, and absence of clinical signs of relapse. The temporal association with poppy seed consumption, together with resolution on repeat testing, supports the conclusion that dietary exposure was responsible.

False-positive opioid screens can have significant clinical implications, including unnecessary treatment adjustments, increased monitoring, or erosion of trust between patients and clinicians. Detailed dietary histories, patient counseling, and repeat testing when unexpected results occur can help distinguish between true non-adherence and food-related effects.

**Conclusion:**

Poppy seed consumption can cause transient false-positive opioid urine screens in patients receiving opioid substitution therapy, potentially leading to clinical confusion and unnecessary interventions. Clinicians should consider dietary sources when interpreting unexpected opioid results, take detailed dietary histories, and provide patient education.

